# The diagnostic value of chest X‐ray scanning by the help of Artificial Intelligence in Heart Failure (ART‐IN‐HF)

**DOI:** 10.1002/clc.24105

**Published:** 2023-08-31

**Authors:** Ahmet Celik, Ali Orcun Surmeli, Mustafa Demir, Kaan Esen, Ahmet Camsari

**Affiliations:** ^1^ Department of Cardiology Mersin University Medical Faculty Mersin Turkey; ^2^ Department of Radiology Mersin University Medical Faculty Mersin Turkey

**Keywords:** artificial intelligence, chest X‐ray, heart failure, heart failure with preserved ejection fraction

## Abstract

**Background:**

Typical signs of heart failure (HF), like increased cardiothoracic ratio (CTR) and pleural effusion, can be seen on X‐ray. Artificial Intelligence (AI) can help in the early and quicker diagnosis of HF.

**Objectives:**

The study's goal was to demonstrate that the AI interpretation of chest X‐rays can assist the clinician in diagnosing HF.

**Methods:**

Patients older than 45 years were included in the study. The study analyzed 10 100 deidentified outpatient chest X‐rays by AI algorithm. The AI‐generated report was later verified by an independent radiologist. Patients with CTR > 0.5 and pleural effusion were marked as potential HF. Flagged patients underwent confirmatory tests, and those labeled as negative also underwent further investigations to rule out HF.

**Results:**

Out of 10 100, the AI algorithm detected 183 (1.8%) patients with increased CTR and pleural effusion on chest X‐rays. One hundred and six out of 183 underwent diagnostic tests. Eighty‐two (77%) out of 106 were diagnosed with HF according to current guidelines. From the remaining 9917 patients, 106 patients were randomly selected. Nine (8%) out of them were diagnosed with HF. The positive predictive value of AI for diagnosing HF is 77%, and the negative predictive value is 91%. More than half (54.9%) of newly diagnosed patients had HF with preserved ejection fraction.

**Conclusion:**

HF is a risky condition with nonspecific symptoms that are difficult to diagnose, especially in the early stages. Using AI assistance for X‐ray interpretation can be helpful for early diagnosis of HF especially HF with preserved ejection fraction.

## Graphical abstract

Artificial Intelligence (AI) may help in the early and quicker diagnosis of heart failure (HF). Patients with cardiothoracic ratio (CTR) >0.5 and pleural effusion on chest X‐rays were marked as potential HF by AI. Potential HF patients underwent diagnostic tests such as natriuretic peptides and echocardiography. AI algorithm detected 183 (1.8%) patients with increased CTR and pleural effusion on chest X‐rays of 10,100 patients. 77% of AI‐positive patients were diagnosed with HF. Eight percent of AI‐negative patients were diagnosed with HF. The positive predictive value of AI for diagnosing HF is 77%, and the negative predictive value is 91%. The majority of newly diagnosed HF patients had non‐reduced ejection fraction.

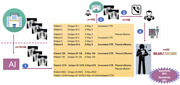

## 1 INTRODUCTION

Heart failure (HF) is a clinical syndrome due to a structural or functional disorder that results in elevated intracardiac pressures and/or inadequate cardiac output at rest and/or during exercise.

HF is a serious heart condition with significant prevalence. Due to the increased survival of coronary vascular disease patients, the prevalence of HF is rising worldwide.^1^

The mortality of HF patients remains high and can range from 5% to 15%, even during hospitalization.^2^ The high hospitalization rate also adds to the economic burden making HF a leading cause of hospitalization for patients older than 65 years of age.^3^ HF has high mortality even after, in time hospitalization. According to some studies, the mortality can be as high as 10.4% within 30 days of hospitalization. The mortality rate doubles to 22% in 1 year and quadruples to 42.3% in 5 years.^4^ The improvement of therapies and medication has improved survivability, but still, a lot needs to be done to reduce high mortality rates.

Patients of HF often present with dyspnea as one of the chief complaints. A chest X‐ray is the first modality of imaging as this is low‐cost, noninvasive, and helps differentiate between cardiac and pulmonary reasons of dyspnea.^5^ HF has many signs that can be picked up on X‐ray; these include cardiothoracic ratio (CTR) > 50%, pulmonary congestion, bilateral pleural effusions, Kerley B lines, hilar haziness, cephalization of pulmonary vessels, and so forth.^6^ Bilateral pleural effusion results in difficulty in breathing and is one of the indicators of HF. However, an increased CTR may not be present in patients with HF.^7^ A study on the utility of X‐rays in HF patients demonstrated that cardiomegaly and redistribution were good indicators of reduced HF.^7^ However, they were not sufficient findings for the diagnosis of HF.^7^ Additionally, in a multicentre study, only cardiomegaly was found to have a sensitivity of more than 50%.^8^ Thus, cardiomegaly or an increase in CTR, along with the presence of congestion in the form of pleural effusion, can be identified, and an Artificial Intelligence (AI) model can be used to identify potential HF patients.

Deep Learning has been used to help assess HF using different types of clinical information.^9^ However, most of these studies have looked at clinical data, and not much has been done to analyze image analysis, especially X‐rays, to determine HF. X‐ray is an inexpensive diagnostic tool that is widely available across the world. Through this study, we want to prove that deep learning models can analyze X‐rays and help identify potential HF cases based on increased CTR and the presence of pleural effusion together on X‐rays. Using the power of AI and the universal availability of X‐Ray will help in the better and quicker diagnosis of HF, even in low‐resource settings, which might lack sophisticated diagnostic tools to diagnose HF. This combination of AI and X‐ray can help reduce mortality and early diagnosis of HF in people on a broader scale.

A rapid and accurate HF diagnosis is of utmost importance to decrease the patient's mortality and reduce the medical expenditure. Our project aims to identify potential HF patients by detecting increased CTR and pleural effusion on their chest X‐rays with the help of AI scanning and thus assist in the quicker diagnosis of HF.

## 2 METHODS

The study was conducted as a single‐center prospective study where X‐rays of eligible patients were analyzed from January to September 2021. Ethics approval (2021/767) was obtained before the initiation of the study from Mersin University Ethics Committee members. During the study period, the AI and expert radiologists analyzed chest X‐rays of subjects admitted to the outpatient departments of Mersin University Medical Faculty Hospital between January 1, 2021, and September 30, 2021. Subjects older than 45 years were included in the study. The patients who were admitted to outpatient clinics of cardiology and cardiovascular surgery were excluded. The patients who were admitted to emergency department, hospitalized patients, and patients with history of HF were excluded from the study. An AI algorithm analyzed the chest X‐rays of selected subjects. AI positive had findings of increased CTR and pleural effusion. Subjects with increased CTR (>0.5) and bilateral pleural effusion were marked as potential HF patients on their X‐rays. These patients were then invited to the cardiology outpatient department for further, definitive HF diagnostic tests to confirm the diagnosis of HF. They underwent physical examination, natriuretic peptides, and transthoracic echocardiography tests in addition to initial Chest X‐rays. The diagnosis of HF were done according to the “2021 ESC Guidelines for the Diagnosis and Treatment of Acute and Chronic HF.^10^ All examinations were performed by an experienced echocardiographer with a Vivid E9 machine (General Electric) equipped with a 3.5 MHz transducer, echocardiographic images were recorded during three cardiac cycles at the end of expiration. Linear dimensions of left ventricular (LV) and left atrium were measured in parasternal long axis. volumes and left ventricular ejection fraction (LVEF) were measured in the apical four‐ and apical two‐chamber views by biplane method of disks summation.

Subjects (randomly sampled) who did not show increased CTR and pleural effusion on their chest X‐rays were also invited to rule out HF diagnosis in them. We used qXR (Qure.ai) for chest X‐rays analysis of selected patients. First, all the DICOM (Digital Imaging and Communications in Medicine) images of X‐ray images were deidentified. Then, these deidentified DICOM images were imported into the system to be analyzed by qXR. These deidentified X‐rays were then analyzed by an AI algorithm (qXR) and were later verified by an independent radiologist from the hospital with experience in thoracic imaging. A final report was generated for each case. Further statistical analyses were done based on the radiologist's report and qXR findings.

The qXR has been trained on 2.3 million+ chest X‐rays. A Deep Learning model called convolutional neural networks (CNNs) was used to develop qXR.^11^ The ResNet^12^ and Squeeze‐and‐Excitation Networks^13^ have been utilized to develop the qXR architecture. The chest X‐rays were resized, down‐sampled to a standard size, and image normalization was performed before training the model with the data. Multiple models were trained to detect each abnormality, and model ensembling was performed to improve the final deep‐learning model robustness, generalization convergence, and incremental gains.”

### 2.1 Statistical analysis

Statistical analysis was performed using IBM SPSS statistics version 21 (IBM Corporation). Continuous data are presented as mean or median for normal, nonnormally distributed variables, respectively, and categorical data are shown as numbers (percentages). To evaluate the diagnostic performance of AI for HF, sensitivity, specificity, positive predictive value (PPV), negative predictive value (NPV), positive likelihood ratio, negative likelihood ratio, accuracy, and diagnostic odds ratio (OR) analysis were done with 95% confidence Interval. A *p* < .05 was accepted as statistically significant from a statistical perspective.

## 3 RESULTS

**Figure 1:**
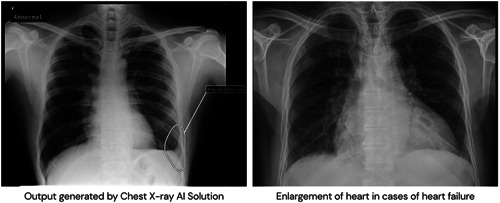
The first chest X‐ray (left side) shows the qXR‐generated output of an HF case. It has secondary capture where AI has marked the abnormality on the X‐ray. In this X‐ray, there is an enlargement of the heart and also shows a pleural effusion. Secondary capture localized the pleural effusion on the X‐ray which is marked by a circle. This makes it easy for the clinicians reading the X‐ray to locate and quantify the effusion. Secondary capture and marking help clinicians to locate the abnormalities and quicker diagnosis of HF. These abnormalities can be followed over time through sequential X‐rays, and the changes can be tracked. Since there are findings (e.g., pleural effusion) in the chest X‐ray, the qXR has labeled the X‐ray as “abnormal.” The second X‐ray (X‐ray on the right side) shows an enlarged heart which is one of the signs of HF. HF, heart failure.

**Figure 2:**
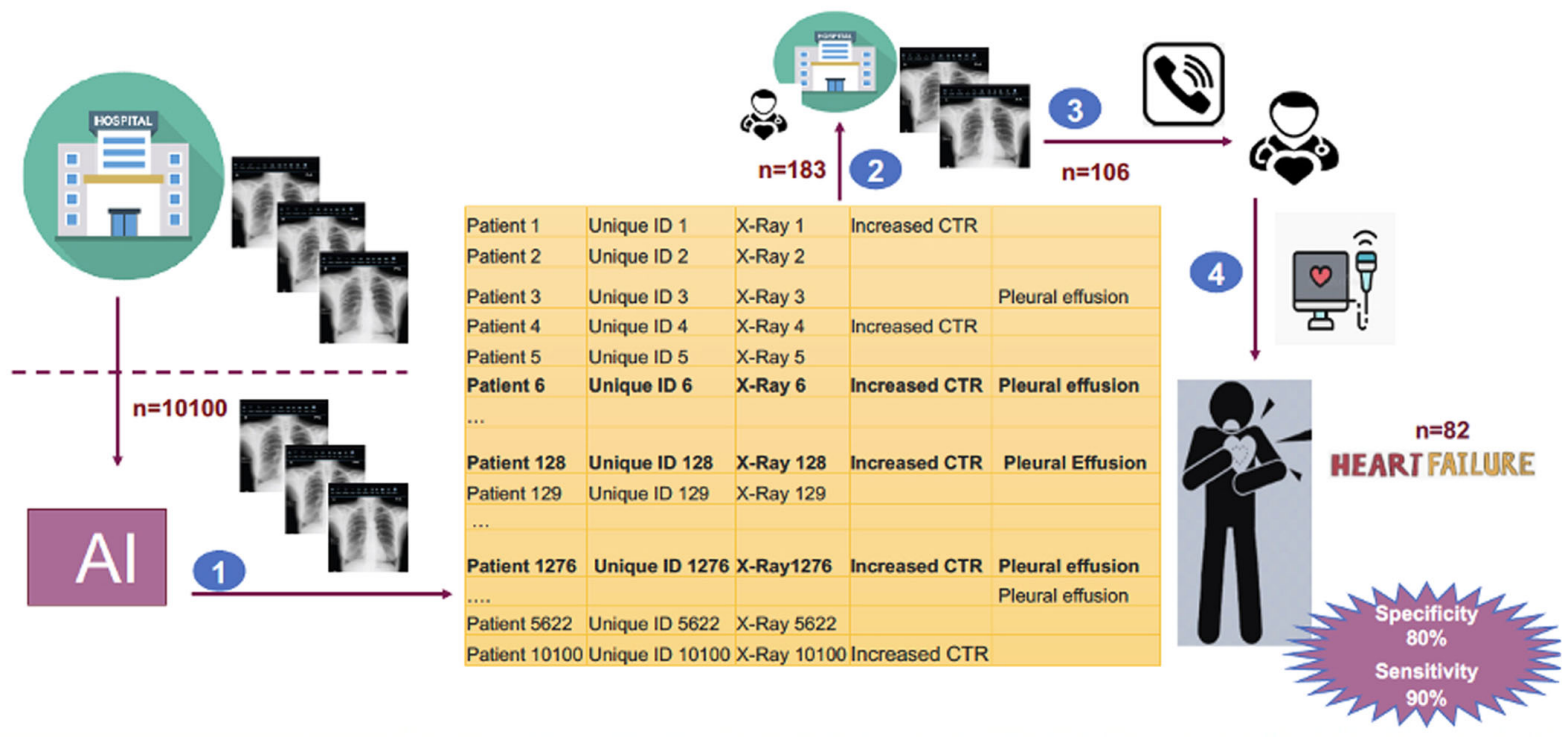
(Central illustration). ART‐IN‐HF study design & steps involved in study. ART‐IN‐HF, Artificial Intelligence in Heart Failure.

Based on the eligibility criteria, anonymous chest X‐rays of 10 100 patients were uploaded on the qure.ai platform. Figure 1 showed qXR generated output using chest X‐ray. AI scanned all these X‐rays and detected 183 patients (1.8%) with increased CTR and pleural effusion on their chest X‐rays. To confirm these findings, we invited all 183 patients to the cardiology clinic for definitive HF diagnostic tests but 106 of them accepted to undergo diagnostics tests. Out of 106 patients, about 77% (82) were diagnosed with HF according to the 2021 ESC HF guidelines. We then analyzed the data of the remaining 9917 subjects without increased CTR and pleural effusion on their chest X‐rays. We randomly selected 106 subjects among them and found that nine of these 106 subjects (8%) were diagnosed with HF (Figure 2).

The baseline characteristics of these patients were summarized in Table 1 as AI positive and AI negative groups. The baseline cardiac and noncardiac parameters of AI‐flagged patients were significantly different than those not flagged by AI. The AI‐flagged patients had lower average LVEF, larger left ventricular diastolic dimension, and thicker interventricular septum diameter on transthoracic echocardiography (Supporting Information: Figure S1). N‐terminal pro‐B‐type Natriuretic Peptide (NT‐proBNP) was more than 10 times higher in flagged patients as compared to non‐flagged patients. The estimated glomerular filtration rate (eGFR) was lower in flagged patients at 65 mL/min/1.73 m^2^, and as a result, serum creatinine in flagged patients was higher at 1.28 as compared to only 0.81 in nonflagged patients. Flagged patients were slightly older than nonflagged patients and a had higher percentage of coronary artery disease, previous myocardial infarction, diabetes, and hypertension.

**Table 1:** The baseline characteristics of study patients.

	AI(+) (*n* = 106)	AI(−) (*n* = 106)	*p* Value
LVEF %	49 ± 12	57 ± 7	<.001
LVDD (cm)	4.8 ± 0.7	4.5 ± 0.5	.002
IVSD (cm)	1.2 ± 0.2	1.1 ± 0.1	.002
PWDD (cm)	1.1 ± 0.1	1.0 ± 0.1	.02
NT‐proBNP	2329 ± 3054	212 ± 328	<.001
eGFR (mL/min/1.73 m^2^)	65 ± 28	85 ± 22	<.001
Serum creatinine (mg/dL)	1.28 ± 1.16	0.81 ± 0.32	<.001
Hemoglobin (g/dL)	11.5 ± 1.9	12.8 ± 1.9	<.001
Age (years)	71 ± 10	66 ± 11	.001
DM %	33	25.5	.291
HT %	56.6	48.1	.271
CAD %	44.3	22.6	.001
MI %	13.2	7.5	.260
Chemo exp (%)	9.4	20.8	.03

Abbreviations: AI, artificial intelligence; CAD, coronary artery disease; Chemo exp, Chemotherapeutic drug exposition; DM, diabetes mellitus; eGFR, estimated glomerular filtration rate; HT, hypertension; IVSD, interventricular septum diameter; LVDD, left ventricular diastolic dimension; LVEF, left ventricular ejection fraction; MI, myocardial infarction; NT‐proBNP, N‐terminal pro‐B‐type natriuretic peptide; PWDD, posterior wall thickness diastolic diameter.

**Figure 3:**
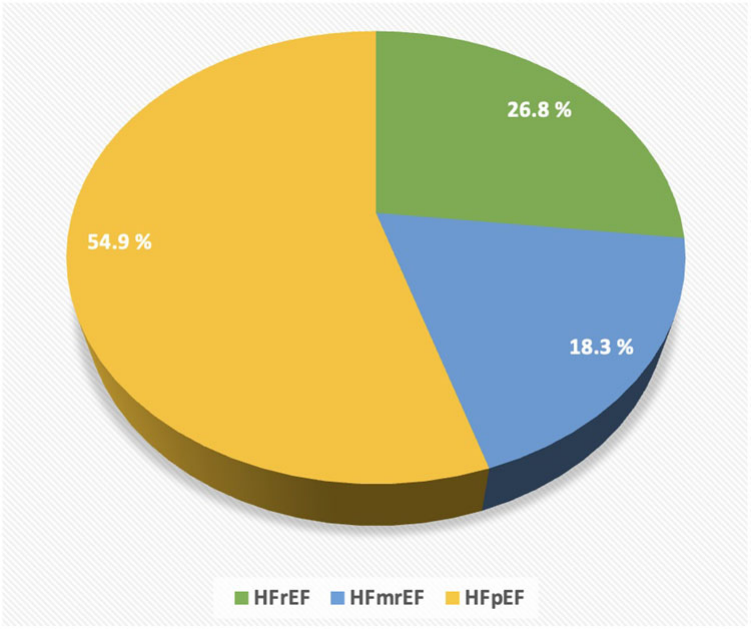
The type of heart failure in newly diagnosed patients with HF by AI. AI, artificial intelligence; HF, heart failure.

An accuracy of 84% was found in diagnosing HF using the chest X‐ray images that AI analyzed. The diagnostic value of AI for HF is summarized in Table 2. The demographic, laboratory, and echocardiographic properties of 82 newly diagnosed HF patients are summarized in Supporting Information: Table S1. About a quarter of newly diagnosed HF patients, 22 (26.8%) of 82, had HF with reduced ejection fraction (HFrEF). Almost one‐fifth (15 (18.3%) of 82 patients) had HF with mildly reduced ejection fraction (HFmrEF), and more than half (45 (54.9%) of 82 patients) had HF with preserved ejection fraction (HFpEF) (Figure 3).

**Table 2:** Diagnostic value of AI in HF according to the chest X‐rays of subjects.

Evaluation metrics	Mean CI (point estimate)	%95 CI lower	%95 CI upper
Sensitivity	0.90	0.84	0.96
Specificity	0.80	0.73	0.87
Positive predictive value	0.77	0.69	0.85
Negative predictive value	0.91	0.86	0.96
Likelihood ratio+	4.54	3.15	6.54
Likelihood ratio−	0.12	0.07	0.23
Accuracy	0.84	0.80	0.89
Diagnostic odds ratio	36.82	16.21	83.66

Abbreviations: AI, artificial intelligence; CI, confidence interval; HF, heart failure.

## 4 DISCUSSION

HF is a complex, risky cardiac condition that is difficult to identify in the initial stages. In addition, HF presents nonspecific symptoms like cough and dyspnea that are confused with pulmonary disorders, thus causing a delay in diagnosis and increased morbidity. Among the three types of HF, HFpEF (also known as diastolic HF) is the most difficult to diagnose due to the lack of apparent signs and symptoms of HF.^14^ However, HFpEF prevalence is increasing with an increasing number of HF cases across the globe. It constitutes almost 50% of all new HF cases, and its proportion in comparison to HFrEF is increasing.^15^, ^16^

HF due to reduced EF is easier to diagnose as the symptoms are more apparent and the patient presents earlier. However, it is challenging to analyze, especially in outpatient settings, in the absence of overt symptoms.^17^, ^18^ Multiple attempts have been made to diagnose HFpEF, including various scoring systems.^19^ These scoring systems consider a combination of echocardiography values, comorbidities, biomarkers inclusions, hemodynamic assessment, and exercise stress testing values.^14^, ^20^, ^21^, ^22^ However, these scoring systems are challenging to apply, especially in low‐resource and remote settings.

There are invasive tests like natriuretic peptides that can indicate HF. Some noninvasive diagnostic modalities like echocardiography and exercise stress tests also help diagnose. However, these expensive tests are difficult to perform in multiple settings, especially in low‐resource or nontertiary setups, and are not widely available.

X‐ray is a noninvasive diagnostic modality that is widely available worldwide and is low‐cost. X‐rays are the first imaging modality that is done when patients present with symptoms of HF to differentiate the cardiac from the pulmonary cause. There are HF signs that can be seen on X Rays like increased CTR, Kerley B lines, and pleural effusion that can be subtle, especially in HFpEF, and are difficult to identify on an X‐ray. However, with proper training, an AI algorithm can pick these signs and help in identifying potential HF patients early in the disease state. The use of AI can assist with the shortage of trained clinicians who can diagnose HF on X‐rays in low‐resource and remote settings.

ART‐IN‐HF project is the pioneering initiative that used chest X‐ray analysis by AI for the early diagnosis of HF. This was done by detecting increased CTR and pleural effusion together on the X‐rays of potential HF cases. Diagnostic accuracy of X‐ray analysis by AI for HF in patients with pleural effusion and increased CTR was found in 183 patients (1.8%). AI will be helpful for detecting HF using chest X‐ray scans in undiagnosed HF patients, especially for difficult‐to‐diagnose HFpEF patients. Most newly diagnosed HF patients (73.2%) have HF with nonreduced EF. Data suggests that the undiagnosed HF patients in the population likely have HF with nonreduced HF.

Although it is accepted that the proportion of patients with HFpEF is equal to that of HFrEF (approx. 50% of all HF patients have HFpEF, and 50% of them have HFrEF), in the European Society of Cardiology Heart Failure Long‐Term Registry, the ratio of patients with HFpEF is 16%, and the proportion of patients with HFmrEF was 24.2%. SELFIE‐TR^23^ study showed that the proportion of patients with HFpEF is only 7.3%, and 16.7% of them had HFrEF. However, in the ART‐IN‐HF trial, we showed that 54.9% of the undiagnosed HF population have HFpEF. The ART‐IN‐HF trial demonstrates the effectiveness of AI triaging of CXR to diagnose HF. Furthermore, the trial was conducted on a population who had never visited the cardiology department, which signifies the potential of AI triaging of CXR in identifying HF patients. The undefined proportion of HFpEF patients might appear in larger populations. AI has been used previously to help in the screening of HFpEF.^24^ This study adds to the published evidence of the AI' utility for diagnosing HFpEF.AI has assisted busy clinicians in using the diagnostics modalities like echocardiography for the diagnosis of cardiological conditions.^25^ This not only helps in quicker diagnosis but also helps in better confirmation of findings. In this study, we would like to extend the utility of AI to another diagnostic modality, X‐ray, for early diagnosis of HF.

An increase in awareness of HF without reduced LVEF can be beneficial for both patients and physicians. This will help in early diagnosis and initiation of treatment, especially in patients who have risk factors for developing HF (e.g., diabetes, hypertension, etc.). In addition, early diagnosis of HF will help in reducing morbidity and mortality of patients as well as cost savings by reducing complications. This study demonstrates that AI assistance can be helpful for the early detection of HF using X‐rays, even in non‐symptomatic or minimally symptomatic patients.

Our study is one of the first attempts to use deep learning to read X‐rays and assist in the diagnosis of potential HF cases. Our study adds to the evidence and demonstrates that AI can be used for the early detection of HF cases using X‐rays, even in non‐symptomatic or minimally symptomatic patients. The promising results of this study are encouraging and warrant a multicentre prospective clinical trial to learn more about the impact of AI on treatment outcomes and to create a new protocol for screening of HF using AI.

### 4.1 Study limitations

The study was a single‐site study; however, a multisite study might be more generalizable. The inclusion criteria included only 45 years and older. However, HF can occur in younger patients also. The proportion of participants who underwent confirmatory tests could have been larger, which could have shown a much greater number of HF cases. Study results being at the tertiary center might not be applicable at the smaller or nontertiary centre. The study population was mainly from a particular geographic location, that might have influenced the final results.

### 4.2 Perspectives

Diagnosis of HF for clinicians is not easy. Diagnosis becomes even more difficult without the provision of tests like proBNP. HF signs can be picked up on chest X‐rays. The use of AI has been on the rise in the field of cardiology. This study demonstrated that AI could help in the diagnosis of HF. There are many potential clinical implications of this study. This study opens many opportunities for generating newer medical knowledge where the early changes in HF can be tracked through AI by sequential X‐ray over time and can play a vital role in newer clinical evidence generation.

The discovery of newer cases early in the disease process might result in a change in documented prevalence of HFpEF in the general population. This potential change in prevalence might result in a change in the guidelines for screening and management protocols of HF.

Using AI in HF diagnosis will also result in better patient care as more HFpEF patients can be identified earlier in the process and, appropriate clinical care can be initiated before the onset of symptoms. This will reduce chances of complications in patients, the morbidity and burden of HF disease in the population.

Early diagnosis of HFpEF with other comorbidities will help identify patients who need active interventions like cardiac procedures. This might result in newer modalities of treatment and surgical intervention for patients with specific comorbidities in addition to HF.

This study can become a catalyst for more research in early diagnosis of HF using machine learning or AI. In addition, this can become a precursor of multicentre clinical trials where even bigger numbers of patients with multiple comorbidities might be part of the trial. Such trials can generate medical evidence that can change the guidelines of HF management. These can also be instrumental in newer specific scientific evidence for the discovery of newer drugs or newer surgical modalities to manage HF.

## CONFLICT OF INTEREST STATEMENT

The authors declare no conflict of interest.

## Supporting information

Supplement Figure 1. The comparison of subjects according to AI flagged or non‐flagged.

Supporting information.

## Data Availability

We assure data availability.
