# Supplementary figures and images for: The diagnostic value of chest X‐ray scanning by the help of Artificial Intelligence in Heart Failure (ART‐IN‐HF)
Source: Clin Cardiol. 2023 Aug 31;46(12):1562–8. doi: 10.1002/clc.24105 (PMC10716309; doi:10.1002/clc.24105)

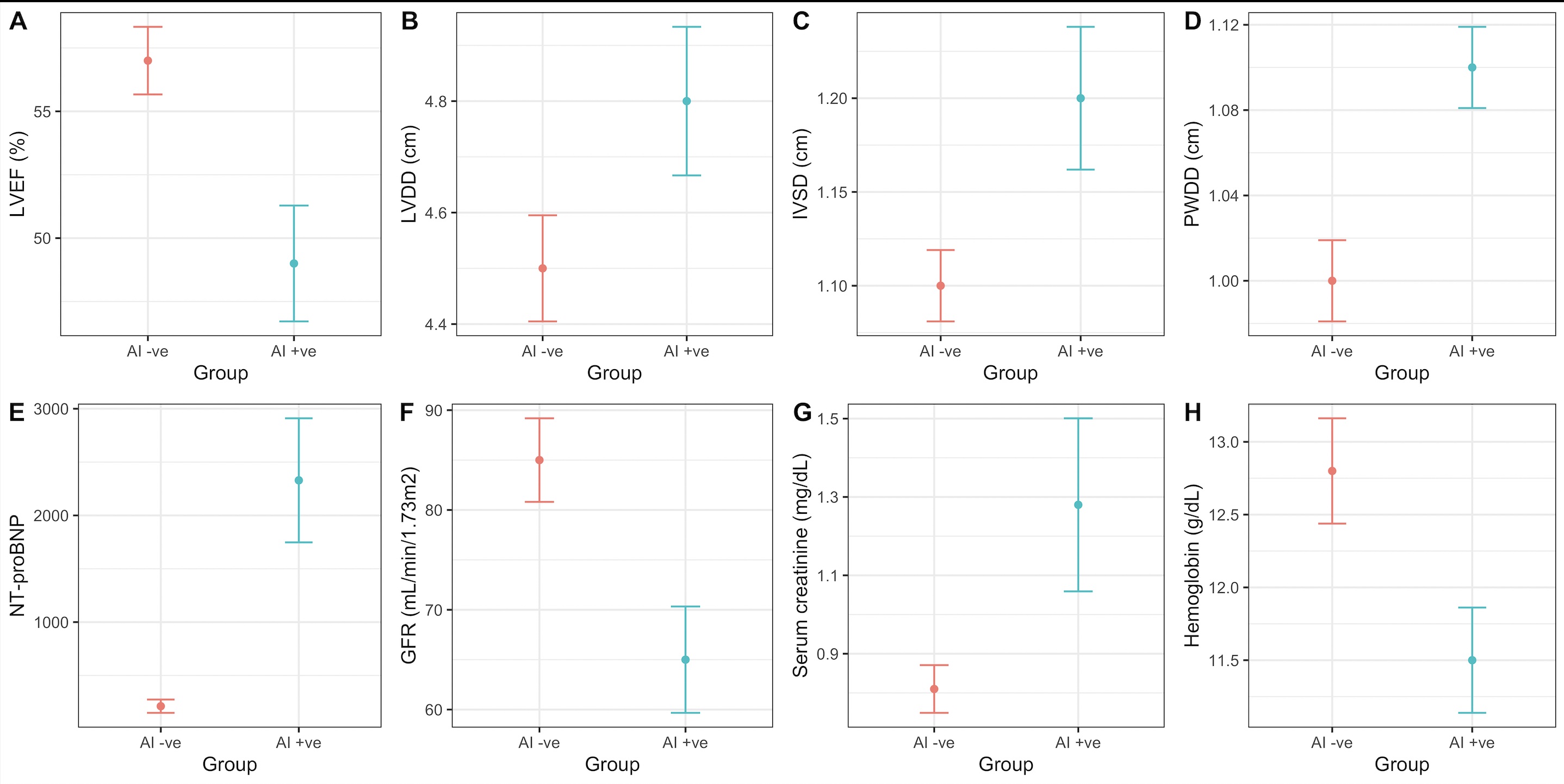

Supplement: Supplementary file 1 — Supplement Figure 1. The comparison of subjects according to AI flagged or non‐flagged. [file CLC-46-1562-s001.jpg]
