# Supplementary material for: The diagnostic value of chest X‐ray scanning by the help of Artificial Intelligence in Heart Failure (ART‐IN‐HF)
Source: Clin Cardiol. 2023 Aug 31;46(12):1562–8. doi: 10.1002/clc.24105 (PMC10716309; doi:10.1002/clc.24105)
Supplement: Supplementary file 2 — Supporting information. [file CLC-46-1562-s002.docx]

**Supplement Table 1 Characteristics of the patients with newly diagnosed HF by AI (n=82)**

| **Variables** | **Distributions** |
| --- | --- |
| ***Age (years)*** | 70±10 |
| ***Ischemic heart disease (n, %)*** | 38, 46.3 % |
| ***Diabetes mellitus (n, %)*** | 30, 36.6% |
| ***Hypertension (n, %)*** | 48, 58.5% |
| ***History of MI (n, %)*** | 13, 15.9% |
| ***Chemotherapeutic drug exposition (n, %)*** | 7, 8.5% |
| ***NT-proBNP (pg/mL)*** | 2979±3193 |
| ***Serum creatinine (mg/dL)*** | 1.37±1.28 |
| ***eGFR (mL/min/1.73m^2^)*** | 63±28 |
| ***Na (mEq/L)*** | 138±4 |
| ***K (mEq/L)*** | 4.4±0.5 |
| ***Hb (g/dL)*** | 11.5±1.8 |
| ***WBC (x10^Ù^3/mL)*** | 8.1±3.9 |
| ***LVEF %*** | 46±12 |
| ***LVDD (cm)*** | 4.9±0.8 |
| ***IVSD*** | 1.2±0.3 |
| ***PWDD*** | 1.1±0.1 |

HF: heart failure, AI: artificial intelligence, MI: myocardial infarction, NT-proBNP: N-terminal pro B-type natriuretic peptide, eGFR: estimated glomerular filtration rate, Na: sodium, K: potassium, Hb: hemoglobine, WBC: white blood cell, LVEF: left ventricular ejection fraction, LVDD: left ventricular diastolic diameter, IVSD: interventricular septum diastolic diameter, PWDD: posterior wall diastolic diameter
